# Supplementary material for: Neighbourhood-level socio-demographic characteristics and risk of COVID-19 incidence and mortality in Ontario, Canada: A population-based study
Source: PLoS One. 2022 Oct 20;17(10):e0276507. doi: 10.1371/journal.pone.0276507 (PMC9584389; doi:10.1371/journal.pone.0276507)
Supplement: S1 Table — (PDF) [file pone.0276507.s001.pdf]

van Ingen et al. Neighbourhood-level socio-demographic characteristics and risk of COVID-19 incidence and mortality in Ontario, Canada: a population-based study

Supplementary Table 1. Neighbourhood-level socio-demographic characteristic variable descriptions.

Neighbourhood-level socio-demographic characteristic	Description
**Immigration and race**	
All immigrants	'Immigrant' refers to a person who is, or who has ever been, a landed immigrant or permanent resident of Canada.[a]
Recent immigrant	Any person who has immigrated to Canada within five years of the census date (i.e. 2011-2016). [a]
Visible Minority Status	'Visible minority' refers to whether a person belongs to a visible minority group as defined by the *Employment Equity Act* and, if so, the visible minority group to which the person belongs. The *Employment Equity Act* defines visible minorities as "persons, other than Aboriginal peoples, who are non-Caucasian in race or non-white in colour". Statistics Canada further categorizes the visible minority population into the following population groups: Arab, Black, Chinese, Filipino, Japanese, Korean, Latin American, Southeast Asian, South Asian and West Asian, multiple visible minorities, and visible minorities not included elsewhere.[b]
Black	Black visible minority population group.
East/Southeast Asian	Consisting of Chinese, Filipino, Korean, Japanese, and Southeast Asian (e.g., Vietnamese, Cambodian, Laotian, Thai, etc.) visible minority population groups. Population groups were combined according to the Government of Ontario's Anti-Racism Data Standards.[c]
Latin American	Latin American visible minority population group.
Middle Eastern	Consisting of Arab, and West Asian (e.g., Iranian, Afghan, etc.) visible minority population groups. Population groups were combined according to the Government of Ontario's Anti-Racism Data Standards. [c]
South Asian	South Asian (e.g., East Indian, Pakistani, Sri Lankan, etc.) visible minority population group.
**Housing**	
Average household size	'Household size' refers to the number of persons in a private household.[d]
Multigenerational families	Multigenerational families are households with at least one person living with a child and a grandparent.
Unsuitably crowded housing	Describes whether a dwelling has enough bedrooms for the size and composition of the household, based on the age, sex, and relationships among household members. [e] The number of bedrooms is based on the National Occupancy Standard, which requires the following:<br>- A maximum of two persons per bedroom.<br>- Household members, of any age, living as part of a married or common-law couple share a bedroom with their spouse or common-law partner.<br>- Lone parents, of any age, have a separate bedroom.<br>- Household members aged 18 or over have a separate bedroom, except those living as part of a married or common-law couple.

	- Household members under 18 years of age of the same sex share a bedroom, except lone parents and those living as part of a married or common-law couple.<br>- Household members under 5 years of age of the opposite sex share a bedroom if doing so would reduce the number of required bedrooms. This situation would arise only in households with an odd number of males under 18, an odd number of females under 18, and at least one female and one male under the age of 5.
Apartment in duplex or flat	One of two dwellings, located one above the other, may or may not be attached to other dwellings or buildings.[f]
Low-rise apartment	A dwelling unit attached to other dwelling units, commercial units, or other non-residential space in a building that has fewer than five storeys. [f]
High-rise apartment	A dwelling unit in a high-rise apartment building which has five or more storeys.[f]
**Socio-economic status**	
Labour force participation	Population 15+ who were either employed or unemployed. Individuals not in the labour force include students, homemakers, retired workers and persons who could not work because of a long term illness or disability.[g]
Less than high school education	Population 25-64 who has not completed a high (secondary) school diploma or equivalency certificate. [h]
Low income	Refers to the after-tax low income cut-offs (LICO)) measured at the economic family level (includes all family members living in a shared dwelling). Income cut-offs vary by size of family size and across communities of different sizes.[i]
Unaffordable housing	Households (renters, owners, and total) spending 30% or more of total household income on shelter expenses. Shelter expenses include payments for electricity, oil, gas, coal, wood or other fuels, water and other municipal services, monthly mortgage payments, property taxes, condominium fees and rent.[j]

Sources:

[a] Statistics Canada. Dictionary, Census of Population, 2016: Immigrant status. 2017. https://www12.statcan.gc.ca/census-recensement/2016/ref/dict/pop148-eng.cfm (accessed 1 Feb 2022).

[b] Statistics Canada. Dictionary, Census of Population, 2016: Visible minority. 2017. https://www12.statcan.gc.ca/census-recensement/2016/ref/dict/pop127-eng.cfm (accessed 1 Feb 2022).

[c] Government of Ontario. *Data Standards for the Identification and Monitoring of Systemic Racism. 2021.* https://www.ontario.ca/document/data-standards-identification-and-monitoring-systemic-racism (accessed 1 Feb 2022).

[d] Statistics Canada. Dictionary, Census of Population, 2016: Household size. 2017. https://www12.statcan.gc.ca/census-recensement/2016/ref/dict/households-menage010-eng.cfm (accessed 1 Feb 2022).

[e] Statistics Canada. Dictionary, Census of Population, 2016: Housing suitability. 2017. https://www12.statcan.gc.ca/census-recensement/2016/ref/dict/households-menage029-eng.cfm (accessed 1 Feb 2022).

[f] Statistics Canada. Structural Type of Dwelling and Collectives Reference Guide, Census of Population, 2016. 2017. https://www12.statcan.gc.ca/census-recensement/2016/ref/guides/001/98-500-x2016001-eng.cfm (accessed 1 Feb 2022).

[g] Statistics Canada. Dictionary, Census of Population, 2016: Labour force status. 2017. https://www12.statcan.gc.ca/census-recensement/2016/ref/dict/pop057-eng.cfm (accessed 1 Feb 2022).

[h] Statistics Canada. Dictionary, Census of Population, 2016: Highest certificate, diploma or degree. https://www12.statcan.gc.ca/census-recensement/2016/ref/dict/pop038-eng.cfm (accessed 1 Feb 2022).

[i] Statistics Canada. Dictionary, Census of Population, 2016: Low-income cut-offs, after tax (LICO-AT). https://www12.statcan.gc.ca/census-recensement/2016/ref/dict/fam019-eng.cfm (accessed 1 Feb 2022).

[j] Statistics Canada. Dictionary, Census of Population, 2016: Core housing need. https://www12.statcan.gc.ca/census-recensement/2016/ref/dict/households-menage037-eng.cfm (accessed 1 Feb 2022).
