# Supplementary material for: Neighbourhood-level socio-demographic characteristics and risk of COVID-19 incidence and mortality in Ontario, Canada: A population-based study
Source: PLoS One. 2022 Oct 20;17(10):e0276507. doi: 10.1371/journal.pone.0276507 (PMC9584389; doi:10.1371/journal.pone.0276507)
Supplement: Attachment — Submitted filename: Neighbourhood and COVID19 - PLOSOne_Response to Reviewers.pdf [file pone.0276507.s002.pdf]

Reviewers' comments:

Comments to the Author

A. Reviewer 1:

Thank you for the opportunity to review this manuscript. The authors describe associations between neighbourhood-level social and demographic characteristics and COVID-19 incidence and mortality rates over a 7-month period at the beginning of the pandemic. This study provides a more granular understanding of the relationship between specific neighbourhood characteristics and COVID-19 outcomes and provides additional evidence for social inequities. The study benefits from access to complete COVID-19 infection and death information over the study period and utilization of multiple Census measures of immigration, race, housing, and socio-economic characteristics. The authors were importantly able to adjust for individual-level age and sex and neighbourhood-level urban/rural variables. Please find additional comments for the authors' consideration to strengthen the manuscript.

General:

1. **Cross-sectional study designs do not generally support the measurement of incidence or relative risk. Can the authors be more specific in their interpretation or provide an explanation for how the study design supports calculation of disease incidence and relative risk?**

Response: We agree that the originally proposed cross-sectional study design was not the most appropriate description of our study design. We used surveillance data that included all new positive SARS-CoV-2 infections (i.e., incidence) over a risk period (January 23 to July 28, 2020) in the defined source population (all residents of Ontario). This study design has been defined as an incidence study by Pierce (https://doi.org/10.1093/ije/dys049). Given the incident outcomes, we estimated incidence relative risks across the study period using Poisson models, offset by population size.

As such, we have made the following changes to the manuscript:

- Title: changed 'cross-sectional study' to 'population-based study'
- Line 24: We now state 'We conducted a population-based study'
- Line 90: We now state 'We conducted a population-based surveillance cohort study'

Abstract:

2. **Lines 31-37: Please include major findings related to housing and socio-economic characteristics in addition to the findings you have presented for neighbourhood-level measures of immigration and race.**

Response: We added the following description to lines 38-43:

“Neighbourhoods with the highest average household size (RR: 1.9, 95%CI:1.7–2.1), proportion of multigenerational families (RR: 1.8, 95%CI:1.7–2.0) and unsuitably crowded housing (RR: 2.1, 95%CI:2.0–2.3) were associated with COVID-19 incidence. Neighbourhoods with the highest proportion of residents with less than high school education (RR: 1.6, 95%CI:1.4–1.8), low income (RR: 1.4, 95%CI:1.2–1.5) and unaffordable housing (RR: 1.6, 95%CI:1.4–1.8) were associated with COVID-19 incidence.”

Materials and Methods:

3. **Line 87: Indicate rationale for dates selected.**

Response: We added the following description to lines 93-94:

“the most recent data available at the time of the study”

4. **Line 104: Indicate Version 7c is for November 2019 postal codes**.

Response: Added line 111.

5. **Line 104: Provide a reference to Census variable definitions (e.g. What is meant by low income or unaffordable housing?)**

Response: Census variables descriptions and references have been added to the new Supplementary table 1 (referred to in the manuscript on line 113). Specifically, low income refers to earning less than the after-tax low income cut-off (LICO), and unaffordable housing is defined as spending more than 30% of income on housing.

6. **Line 113: Indicate four categories and how these map onto binary urban/rural classification**.

Response: We added the following description to line 121:

“Four categories of urban/rural geographic stratification (large urban centre, medium/small urban centre, rural, and remote) were determined by grouping neighbourhoods based on community size, population density, and level of integration with a census metropolitan area or census agglomeration.[20]"

7. **Line 115: Provide rationale for age cut-off values and modelling age as a categorical rather than continuous variable**.

Response: Age was included in our models as a categorical variable to more easily interpret the differences in risk between youth (<15 years of age), working age population (15-64 years old) and seniors (65+ years old). Including age as a continuous variable would have introduced unwanted complexity in interpreting study findings, especially if age acts to increase COVID-19 incidence and mortality risk in a non-log-linear fashion.

Line 124 now states: “Age group (youth (<15 years old), working age (15-64 years old), and older adults (≥65 years old)) and sex…”

8. Line 132: Please confirm whether the authors checked and confirmed that over-dispersion was not an issue such that the Poisson distribution is appropriate.

Response: We added the following explanation to line 143:

“Models were assessed for zero-inflation by comparing the observed number of zeroes with model predicted number of zeroes for all models. No models were found to be underfitting zeroes. Any overdispersion present in outcomes is accounted for by the use of random effects in all models. [21]

9. Line 137: Indicate how 95% confidence intervals were calculated. Did you use robust standard errors?

Response: We have recalculated the 95% confidence intervals using robust standard errors, now noted in line 153-4. The results shown in table 2 have been adjusted to reflect the new confidence intervals. This resulted in only slight changes to the confidence intervals, none of which changed the study results.

10. Line 138: Provide rationale for p10/p90 comparison.

Response: We added the following description to line 150:

“To account for uneven distribution of socio-economic characteristics across neighbourhoods, all model estimates were standardized to show relative risks and 95% confidence intervals of COVID-19 incidence and mortality rates between the 10th (p10) and 90th (p90) percentile of each neighbourhood socio-demographic characteristic.”

Results:

11. Where possible, please clarify whether you are referring to crude or adjusted rates.

Response: Results were thoroughly reviewed and crude and adjusted risk are now indicated throughout the manuscript.

12. Line 174-175: Specifically state what the solid black line and dotted lines represent in Figure 2. Include in the Methods section how the trend line was estimated.

Response: A definition of the solid black line is now included in Figure 2 titles. The Methods section now includes the following information regarding how the trend line was estimated on line 154-6:

“The distribution of socio-demographic characteristics were plotted against COVID-19 incidence for each neighbourhood, along with solid lines representing the model-predicted estimates

(derived using ‘prediction’ package in R) and dashed lines marking p10 and p90 for each predictor's distribution.”

13. **Lines 195-196: Indicate the other characteristics that were also not associated with incidence and/or mortality, particularly after adjustment**.

    Response: Information has been added in lines 206-231.

14. **Lines 198-199: You are also controlling for age and sex as confounders, which are also likely playing an important role here**.

Response: We have adjusted language to reflect all confounders include in statistical adjustments.

Lines 229-31 now reads “In adjusted models the protective association of neighbourhoods with the highest compared to lowest proportion of less than high school education was inversed, indicating an association with increased COVID-19 incidence.”

15. **Lines 211+: Include a summary of results for housing and socio-economic status as well**.

Response: Information has been added in lines 219-231

16. **Figures 2A-C – Include the 95% confidence interval**

Response: We are happy to include if the editors would like this information. However, our intention in figures 2A-C is to show distribution of COVID-19 incidence across Ontario neighbourhoods sorted by prevalence of 18 socio-economic characteristics. The regression lines assist in interpreting the directionality of association. It is our view that 95% confidence intervals are better suited in Table 2, and that adding more information would decrease the legibility of these figures, which already contain considerable amount of information.

17. **Figures 2A-C – Crop x-axes where there are no data points (Middle Eastern, less than high school are notable)**

Response: The figures have been adjusted to crop the x-axis where suggested.

Figures 2A-C – Label all vertical lines (p10, p90)

Response: The figures have been adjusted to label the lines as p10/p90

Discussion:

18. Line 218: Not directly. Your study examines neighbourhood-level characteristics as determinants of inequities but does not examine specific structural barriers. Suggest rewording for accuracy.

Response: Thank you for this comment. We agree and the sentence has been reworded on lines 241-3 to say:

"These findings highlight how neighbourhood-level conditions, which reflect social environments that are influenced by institutional and structural systems (e.g., policy),[22] act as key determinants of COVID-19 inequities."

19. Lines 238-241: If I understand how you constructed your models correctly, your study does not support this conclusion. Did you construct models that included immigration, race, and housing variables together? As I interpreted Table 2, you had separate models for 1) immigration and race, 2) housing, and 3) socio-economic status variables where these groupings were adjusted for age, sex, and urban/rural status. Please clarify.

Response: Thank you for the correction. We have reworded the explanation on lines 263-7 as follows:

"In our study, neighbourhood-level housing characteristics were associated with increasing risk of COVID-19 incidence and mortality. Future research is required to examine the extent to which housing characteristics explain the disproportionate impact of COVID-19 on immigrant, racialized, and low-income communities."

20. Line 270: Please include any limitations associated with using a cross-sectional design.

Response: We added line 319-20 to the limitations section, which states:

"Finally, results from our observational study do not allow for causal relationships to be assessed."

21. Line 271: Were there changes in testing criteria during this time period? If so, please clarify what these changes were.

Response: We have added lines 297-9 which describe changing testing criteria:

"Testing criteria for SARS-CoV-2 during the study period shifted from initially being restricted to identifying cases in returning symptomatic travelers or individuals with direct exposure to a recent traveler, to being broadly expanded to include asymptomatic individuals in May 2020.[46]"

1.

Line 275: I think this misrepresents the findings of Sundaram's study as significant associations for testing and testing positive were indeed found in fully adjusted models, particularly for variables of importance to this study. I would suggest reconsidering the

role of selection bias in your findings.

Response: We agree our representation of the study findings were too general. We have added the following description to line 301-308:

"An Ontario study undertaken concurrently with the current study's period of observation found decreased odds of having been tested for COVID-19 (i.e. communities with higher percentages of lower income and visible minorities) and increased odds of having received a positive COVID-19 diagnosis (i.e., increase quintile of people per dwelling and with limited education attainment) in models adjusted for age, sex, underlying health conditions, previous health care, public health region, environment and area-based social determinants of health.[47] The resulting under detection suggest the associations between neighbourhood socio-demographic factors and COVID-19 incidence and mortality in our study are likely conservative."

22. **Line 280: I am not sure what you mean by the term 'dilute'. Is there an alternate term that can be used?**

Response: We removed the term dilute and instead highlight the differences between area and individual measures. This section now reads:

"Additionally, our area-level findings should not be interpreted at the individual-level, as individual cases may not reflect the characteristics of the neighbourhoods they live in. Previous Canadian studies comparing individual and area-level measures have shown that even with relatively poor agreement between measures, area-level measures may be describing important community-level effects that contribute to health inequities.[48]"

Conclusion:

23. **Line 296: Include immigration as a key neighbourhood characteristic.**

Response: Immigrant was added to the description. Line 327-330 now reads;

"Neighbourhood socio-demographic factors, including immigration, race, housing and socio-economic status are associated with COVID-19 incidence and mortality in Ontario. These results suggest that culturally safe approaches to engaging with immigrant, racialized and low socio-economic status communities are important public health strategies for reducing COVID-19 inequities."

24. **Line 296: This is the first time you mention poverty and this may not be the most accurate term to use here – perhaps low SES or low income communities would be more appropriate to the study context.**

Response: We agree and have adjusted that term. Please see the changes to the description in comment 23.

Reviewer 2:

This paper was an enjoyable read and a good example of how combing datasets can provide valuable insights for policy makers. The methods used to find associations between neighborhood-level sociodemographic measures and COVID-19 incidence and mortality are well articulated and based on sound statistical methods. The comments below are minor and aim to improve the clarity of the paper for the reader. I recommend publications with minor revision.

25. **Lines 33-37: In abstract, IRRs are difficult to interpret as referent and comparison groups are not clear (what do you mean by high proportion). Including details about the deciles would be helpful (comparing 10p - 90p)**.

Response: We added the following description to lines 28-32;

"Comparing neighbourhoods in the 90th to the 10th percentiles of socio-demographic characteristics, we estimated the associations between 18 neighbourhood-level measures of immigration, race, housing and socio-economic characteristics and COVID-19 incidence and mortality using Poisson generalized linear mixed models."

26. **Line 46: Make it more clear that the South Asian finding was part of the same UK study and make it clear who they have higher odds of mortality compared to**

Response: We have clarified with the following sentence. Lines 50-53It now reads:

"For example, early in the pandemic a United Kingdom (UK) found it was observed that Black adults have four times higher odds of COVID-19 mortality than White adults in the United Kingdom (UK), with; South Asian and mixed ethnicity individuals also have significantly higher odds of mortality.[1]"

27. **Line 64: Perhaps 'context of Ontario' is more appropriate than 'Canadian context', as you highlight the need for jurisdiction specific findings.**

    Response: The suggested change was made on line 70, changing from Canadian to Ontario context.

28. **Line 87: Can you provide a rationale for the dates chosen to define your study period?**

    Response: In designing our study, we started our observation period to coincide with the beginning of the COVID-19 pandemic and follow up to the most recent COVID-19 incidence and mortality available at the time of submission for publication. We have added this detail to lines 90- 94:

"We conducted a population-based surveillance cohort study using data extracted from provincial and local reportable infectious disease surveillance systems, collectively known as the Public Health Case and Contact Management System (CCM) which include all known COVID-19 infections and deaths from Ontario, Canada reported between January 23, 2020 and July 28, 2020, the most recent data available at the time of the study.

29. **Lines 89-92: Do you have a citation to support the statement that the census has poor representation of those groups?**

    Response: We have added lines 96-99 which state:

    "Due to the incomplete enumeration of Indigenous communities living on reserves in the Canadian Census (from which exposure and denominator data are derived), and the exclusion of people living in institutions and congregate living settings from the long-form census, these populations were excluded from this study.[18,19]"

30. **Lines 104-112: Can you include (in appendix or refer to a published source) definitions used for the socio-demographic measures? Many are self explanatory, but some questions I have include what counts as recent immigration? What defines unsuitably crowded housing? What defines low income? What defines unaffordable housing?**

Response: Census variables descriptions and references have been added to the new Supplementary table 1 (referred to in the manuscript on line 113).

Specifically, recent immigration is defined as having immigrated to Canada within five years of the census date (i.e. 2011-2016), unsuitably crowded housing is based on the number of bedrooms for the size and composition of the household (based on the age, sex, and relationships among household members), low income is considered earning less than the after-tax low-income cut-off (LICO), and unaffordable housing is defined as spending greater than 30% of income on housing.

31. **Line 113: Listing the four categories would be helpful.**

Response: Added line 121.

" Four categories of urban/rural geographic stratification (large urban centre, medium/small urban centre, rural, and remote) were determined by grouping neighbourhoods based on community size, population density, and level of integration with a census metropolitan area or census agglomeration.[20]"

32. **Line 141: Information on what if any model diagnostics or assessments of goodness of fit tests were done on the models is needed. Was there over-dispersion?**

Response: We have added the following description to lines 143-6:

"Models were assessed for zero-inflation by comparing the observed number of zeroes with model predicted number of zeroes for all models. No models were found to be underfitting zeroes. Any overdispersion present in outcomes is accounted for by the use of random effects in all models.[21]"

Line 148: How many deaths were in the initial dataset before removing congregate settings? You state 24% of cases were in these settings, but knowing the proportion of deaths provides important context for the overall mortality findings.

Response: We added this information on line 159 of the manuscript, stating that 2,769 deaths were recorded in CCM prior to exclusions.

33. **Line 149: How many Ontario neighborhoods were excluded from the analysis because they were too small? And can you speak to what the characteristics of these small neighborhoods compared to those included in this study? Could this introduce any bias to your results?**

Response: 20 neighbourhoods were excluded due to small populations. The majority of these neighbourhoods had no populations or census data was suppressed by statistics Canada for confidentiality reasons. Additionally, only a very small number of cases were reported for these neighbourhoods (the number cannot be disclosed to prevent residual disclosure of data on Indigenous reserves). For these reasons, we feel that these neighbourhoods represent a limited potential for introducing bias.

We added the following description on line 108:

"Twenty neighbourhoods with small populations (<1,000 people) were excluded due to stability concerns."

34. **Line 189: The term multivariable model causes some confusion as they are interchanged with 'adjusted model' and 'fully adjusted model' in the tables and throughout the paper. I find 'adjusted model' to be the most clear in this case.**

Response: All 'multivariable' and 'fully adjusted' terminology has been changed to 'adjusted model' in the text.

35. **Line 196: I understand what is being referred to when saying the adjusting the model 'reversed' the protective association but I do not know if it is an accurate term to use. Perhaps 'inversed' would be better.**

Response: We have incorporated this suggestion into the manuscript. Line 229-231 now reads:

"In adjusted models the protective association of neighbourhoods with the highest compared to lowest proportion of less than high school education was inversed, indicating an association with increased COVID-19 incidence."

36. **Line 217: I believe your findings do show that neighbourhood level factors are key determinants of COVID-19 inequities, but I am not clear how your findings show 'how structural barriers are acting as key determinants of COVID-19 inequities". I would suggest narrowing the conclusions, or elaborating more on how your findings do support that conclusion (ie. Can you provide an example of a specific structural barrier that acted as a determinant?)**

Response: We have incorporated the suggestion into the manuscript. Line 241-243 now reads:

"These findings highlight how neighbourhood-level conditions, which reflect social environments that are influenced by institutional and structural systems (e.g., policy),[22] act as key determinants of COVID-19 inequities."

37. **Lines 238-241: It sounds like this finding comes from an additional analysis that was not described in the methods and not shown in the results? I believe this analysis is of extreme interest. Being able to show that the socio-demographic factors are intertwined but still independently significant even when controlling for other socio-demographic factors is a major finding. It could also provide important evidence for policy makers (just addressing housing will not erase inequalities). I understand it may be difficult to include given word count limitations, but this analysis would be highly interesting.**

Response: We agree this analysis would be highly interesting. As our initial objective was descriptive in nature, we plan to examine this research question in future analyses through formal mediation analyses. However, we have noted the comment about future research directly in the manuscript on lines 263-266 based on this suggestion. It now reads:

"In our study, neighbourhood-level housing characteristics were associated with increasing risk of COVID-19 incidence and mortality. Future research is required to examine the extent to which housing characteristics explain the disproportionate impact of COVID-19 on immigrant, racialized, and low-income communities."

38. **Lines 270-275: Glad to see you addressed the risk of differential COVID testing biasing the results, as this was a concern of mine reading the paper. You did a very good job addressing this concern**.

Response: Thank you for the comment. We agree this issue is complex and needs to be addressed. Based on comment #22 from Reviewer 1, we have included additional detail on this point. Lines 297-308 now read:

"Testing criteria for SARS-CoV-2 shifted from identifying cases in returning symptomatic travelers or individuals with direct exposure to a recent traveler, to symptomatic individuals and those with occupational exposure to asymptomatic individuals. These changing criteria may have contributed to observed differences in testing patterns between various socio-demographic populations. An Ontario study undertaken during the study period examined in the current study found decreased odds of having been tested for COVID-19 (i.e. communities with higher percentages of lower income and visible minorities) and increased odds of having received a positive COVID-19 diagnosis (i.e., increase quintile of people per dwelling and with limited education attainment) in models adjusted for age, sex, underlying health conditions, previous health care, public health region, environment and area-based social determinants of health.(43) The resulting under detection suggest the associations between neighbourhood socio-demographic factors and COVID-19 incidence and mortality in our study are likely conservative."

39. **Lines 294-295: The claim that socio-demographic factors explain much of the neighbourhood-level variability in COVID-19 needs further evidence. Reading this claim, it sounds like if you were to build a multivariable model with all the socio-demographic measures as explanatory variables, your R-Squared (or pseudo R-Squared) would be greater that 50%. Did you find this? If so, that is great but please elaborate in the results.**

Response: Similar to response 38, we agree this would be an important analysis that we will pursue in a future study. We have changed the conclusion so that it more accurately reflects our current study. Lines 327-328 of our conclusion now reads:

"Neighbourhood socio-demographic factors, including immigration, race, housing and socio-economic status are associated with COVID-19 incidence and mortality in Ontario."

40. **Lines 295-296: The conclusion that "culturally safe approaches to engaging with racialized communities and communities living in poverty, are important public health strategies for reducing COVID-19 inequities" is not fully substantiated by your findings. Your findings have identified the problem (neighborhood-level measures are associated with COVID inequalities) but I believe cannot go as far to suggest what will solve the problem. I believe your research will provide meaningful information to inform public health strategies.**

Response: We agree with the proposed comment, that our study highlights the social inequities in COVID-19 outcomes but does not examine any proposed solutions to solve the problem. However, given the inequities that were uncovered, we feel comfortable with the statement that culturally safe approaches to engaging with communities disproportionately impacted by the COVID-19 pandemic is an important in determining what solutions might be important to pursue towards reducing the observed inequities. We feel strongly that meaningful consultation is the required starting point for any proposed public health strategies.

B. Reviewer 3:

This paper provides a unique look at COVID-19 data at a level not examined in many publications. This work is an important piece of the puzzle in understanding infectious disease mitigation in an outbreak scenario. Congratulations to the authors for producing this quality work

Response: Thank you for the supportive comments.
